# Supplementary material for: Teasing out missing reactions in genome-scale metabolic networks through hypergraph learning
Source: Nat Commun. 2023 Apr 25;14:2375. doi: 10.1038/s41467-023-38110-7 (PMC10130184; doi:10.1038/s41467-023-38110-7)
Supplement: Supplementary file 1 — Supplementary Information [file 41467_2023_38110_MOESM1_ESM.pdf]

Teasing out missing reactions in genome-scale metabolic networks through hypergraph learning

Can Chen *et al.*

Supplementary Note 1. Existing methods of predicting missing reactions

GapFind and GapFill

GapFind and GapFill are two optimization-based algorithms that can be used to identify and fill gaps in GEMs[1]. First, GapFind pinpoints the metabolites in a GEM which cannot be produced under any uptake conditions. Subsequently, GapFill identifies the reactions from a customized multi-organism database that restores the connectivity of these metabolites to the parent network using four mechanisms:

1. Reversing the directionality of one or more reactions in the existing model;
2. Adding reactions from another organism to provide functionality absent in the existing model;
3. Adding external transport mechanisms to allow for importation of metabolites in the existing model;
4. Restore flow by adding intracellular transport reactions in multi-compartment models.

Detailed formulation of the two optimization problems of GapFind and GapFill can be found in[1].

FastGapFill

FastGapFill is an extesion of GapFill. It is the first scalable algorithm capable of efficiently detecting and filling gaps in compartmentalized GEMs[2]. FastGapFill first utilizes the developed FastCore algorithm[3] to compute a near-minimal set of reactions that need to be added to an input GEM to render it flux consistent. Then FastGapFill generates a global model by expanding the compartmentalized metabolic model (i.e., the metabolic model without blocked reactions) by a universal metabolic database (e.g., the KEGG database). Finally, FastGapFill computes a compact flux consistent subnetwork of the global model, which leads to the final gap-filled model.

Matrix boost algorithm

Matrix boost algorithm (BoostGapFill) is the first algorithm in this category that conducts inference jointly in the incidence and adjacency space by performing an iterative completion-matching optimization[4]. Given an incomplete hypergraph $\mathcal{H}$ with n nodes, denote $\mathbf{A} = \mathbf{H}\mathbf{H}^\top \in \mathbb{R}^{n\times n}$ as the adjacency matrix of $\mathcal{H}$. Suppose that the complete adjacency matrix is given by $\mathbf{A} + \Delta\mathbf{A}$, and

it can be decomposed by

$$\mathbf{A} + \Delta\mathbf{A} = \mathbf{A} + [\Delta\mathbf{A}]_{\mathbf{A}} + [\Delta\mathbf{A}]_{\bar{\mathbf{A}}}, \tag{1}$$

where $[\mathbf{X}]_{\mathbf{A}}$ denotes the operation that only keeps the entries of $\mathbf{X}$ at $\mathbf{A}$'s nonempty entries and mask all else, and $[\mathbf{X}]_{\bar{\mathbf{A}}}$ is conversely defined as keeping $\mathbf{X}$ only at $\mathbf{A}$'s empty entries. Define $\mathbf{A}+[\Delta\mathbf{A}]_{\mathbf{A}} = \mathbf{A}^+$ and $[\Delta\mathbf{A}]_{\bar{\mathbf{A}}} = \Delta\mathbf{A}^-$. BoostGapFill first approximates the empty entries of $\mathbf{A}^+$, denoted by $\Delta\hat{\mathbf{A}}$, with known $\mathbf{A}^+$ (which can be approximated iteratively). The optimization problem is as follows:

$$\min_{\mathbf{\Theta}} \sum_{i<j} \|\mathbf{A}^+_{ij} - y_{ij}\|^2_{\mathrm{F}} + \gamma\mathcal{R}(\mathbf{\Theta}), \tag{2}$$

where $\mathbf{\Theta} = \{w_0, w_i, w_j, v_{if}, v_{jf}\}$ is the set of parameters, $y_{ij} = w_0 + w_i + w_j + \sum_{f=1}^{k} v_{if}v_{jf}$, and $\mathcal{R}$ is a regularizer. After training, $\Delta\hat{\mathbf{A}}$ can be obtained by

$$\Delta\hat{\mathbf{A}}_{ij} = \begin{cases} w_0 + w_i + w_j + \sum_f v_{if}v_{jf} \text{ if } \mathbf{A}^+ = 0, \\ 0 \quad \text{if } \mathbf{A}^+ \neq 0. \end{cases} \tag{3}$$

Let $\mathbf{U} \in \mathbb{R}^{n\times\tilde{m}}$ be the incidence matrix of the candidate hyperlinks of $\mathcal{H}$ and $\mathbf{\Lambda} \in \mathbb{R}^{\tilde{m}\times\tilde{m}}$ be a diagonal indicator matrix of the candidate hyperlinks. In the matching step, BoostGapFill solves the optimization problem as follows:

$$\begin{aligned} &\min_{\mathbf{\Lambda}} \; \|[\mathbf{U}\mathbf{\Lambda}\mathbf{U}^\top]_{\bar{\mathbf{A}}} - \Delta\hat{\mathbf{A}}\|^2_{\mathrm{F}} \\ &\text{subject to } \mathbf{\Lambda}_{pp} = \{0,1\} \text{ for } p = 1,2,\ldots,\tilde{m}. \end{aligned} \tag{4}$$

The optimization problem (4) can be relaxed by making the integer $\mathbf{\Lambda}_{pp}$ continuous within $[0,1]$, which can be solved by subgradient methods. The continuous scores $\mathbf{\Lambda}_{pp}$ can be viewed as soft indicators of the candidate hyperlinks.

BoostGapFill leverages the powerful matrix factorization technique to perform inference in the adjacency space in recovering missing hyperlinks. Yet, it has limited scalibility since the candidate hyperlink set must be present during training. If the candidate hyperlink set becomes extremely large (e.g., the entire BiGG database), the matrix optimization will be difficult (or even impossible) to solve. Moreover, BoostGapFill cannot handle unseen hyperlinks in the test phase.

Coordinated matrix minimization

Coordinated matrix minimization (CMM) is an improved version of BoostGapFill, which introduces a latent factor matrix to significantly simplify the algorithm[5]. CMM alternatively performs non-negative matrix factorization and least square matching in the adjacency space, in order to infer a subset of candidate hyperlinks that are most suitable to fill the target hypergraph. Similarly to BoostGapFill, denote $\mathbf{A} = \mathbf{H}\mathbf{H}^\top \in \mathbb{R}^{n\times n}$ and $\mathbf{U} \in \mathbb{R}^{n\times \tilde{m}}$ as the adjacency matrix of $\mathcal{H}$ and the incidence matrix of the candidate hyperlinks, respectively. Let a non-negative matrix $\mathbf{Q} \in \mathbb{R}^{n\times k}$ be the latent factor matrix ($k \ll n$), and assume that the complete adjacency matrix of the hypergraph is factoried by

$$\mathbf{A} + \mathbf{U}\mathbf{\Lambda}\mathbf{U}^\top \approx \mathbf{Q}\mathbf{Q}^\top, \tag{5}$$

where $\mathbf{\Lambda} \in \mathbb{R}^{\tilde{m}\times\tilde{m}}$ is a diagonal indicator matrix of candidate hyperlinks. To find the missing hyperlinks, CMM solves the following optimization problem by using the expectation–maximization (EM) algorithm:

$$\begin{gathered}\min_{\mathbf{\Lambda},\mathbf{Q}\geq 0} \|\mathbf{A} + \mathbf{U}\mathbf{\Lambda}\mathbf{U}^\top - \mathbf{Q}\mathbf{Q}^\top\|_{\mathrm{F}}^2 \\ \text{subject to } \mathbf{\Lambda}_{pp} = \{0,1\} \text{ for } p = 1,2,\ldots,\tilde{m}.\end{gathered} \tag{6}$$

After relaxing the constraint of $\mathbf{\Lambda}_{pp}$ to be continuous within $[0,1]$, the linear least square problem can be solved very efficiently using off-the-shelf optimization tools such as IBM-CPLEX[6]. Although CMM is simpler than BoostGapFill and exhibits a better performance, it still suffers from the issue of scalibility and cannot handle unseen hyperlinks.

Clique closure-based coordinated matrix minimization

Clique closure-based coordinated matrix minimization (C3MM) is an improved version of CMM, which utilizes the unique characteristic of clique-closure of a hypergraph[7]. C3MM improves CMM by introducing a clique-closure hypothesis into its objective function which significantly hunts down more hyperlinks which are missed by CMM. C3MM first approximates the latent factor matrix $\mathbf{Q} \in \mathbb{R}^{n\times k}$ ($k \ll n$). Suppose that $\tilde{m}$ is the total number of candidate hyperlinks. Given a diagonal indicator matrix $\mathbf{\Lambda}_{\mathbf{U}} \in \mathbb{R}^{\tilde{m}\times\tilde{m}}$ (which can be initialized randomly), C3MM computes

$$\min_{\mathbf{W}\geq 0} \|\mathbf{A} + \mathbf{A}_{\mathrm{CN}} + \mathbf{U}\mathbf{\Lambda}_{\mathbf{U}}\mathbf{U}^\top - \mathbf{Q}\mathbf{Q}^\top\|_{\mathrm{F}}^2, \tag{7}$$

where $\mathbf{A}_{\text{CN}} = \mathbf{A}^2 - \text{diag}(\mathbf{A})$ captures the common neighbor information of the projected graph. Define $\Delta\mathbf{A} = \mathbf{Q}\mathbf{Q}^\top - \mathbf{A}$. To find the missing hyperlinks, C3MM solves the second optimization problem as follow:

$$\begin{aligned} &\min_{\mathbf{\Lambda_U},\mathbf{\Lambda_H}} \|\mathbf{A} - \mathbf{H}\mathbf{\Lambda_H}\mathbf{H}^\top - \mathbf{U}\mathbf{\Lambda_U}\mathbf{U}^\top\|_{\text{F}}^2 + \|\Delta\mathbf{A} - \mathbf{U}\mathbf{\Lambda_U}\mathbf{U}^\top\|_{\text{F}}^2 + \|\mathbf{\Lambda_H}\|_1 \\ &\text{subject to } (\mathbf{\Lambda_U})_{pp} = \{0,1\} \text{ for } p = 1,2,\ldots,\tilde{m} \\ &\qquad (\mathbf{\Lambda_H})_{pp} = \{0,1\} \text{ for } p = 1,2,\ldots,m. \end{aligned} \tag{8}$$

The algorithm solves the two optimization problems alternatively for a certain number of iterations. C3MM has proved to perform well on temporal hyperlink prediction tasks, compared to CMM. However, C3MM has the same issues with BoostGapFill and CMM (i.e., scalability and inability of handling unseen hyperlinks). Therefore, more sophisticated deep learning techniques are needed in order to fix these issues.

Node2Vec-mean

Node2Vec-mean (NVM) is a baseline method for hyperlink prediction with a relatively simple architecture. Given an incomplete hypergraph $\mathcal{H}$ with n nodes, NVM initializes the node features by performing Node2Vec on the clique-expanded graph, where Node2Vec is a random walk-based graph embedding method. Suppose that the feature vector of node v_i is $\mathbf{x}_i$. The feature vector of a hyperlink e_p then can be computed by using a mean pooling function, i.e.,

$$\mathbf{y}_p = \frac{1}{|e_p|} \sum_{v_i \in e_p} \mathbf{x}_i. \tag{9}$$

The final score of e_p can be obtained through a one-layer neural network, i.e.,

$$S_p = \text{sigmoid}(\mathbf{W}_{\text{score}}\mathbf{y}_p + \mathbf{b}_{\text{score}}), \tag{10}$$

where $\mathbf{W}_{\text{score}}$ and $\mathbf{b}_{\text{score}}$ are the learnable parameters in the scoring neural network. During inference, the score $S_e \in [0,1]$ can be viewed as a soft indicator of a unseen hyperlink. However, decomposing a hypergraph into a graph could lose higher-order structural information. Moreover, Node2Vec is computationally expensive when dealing with large graphs.

Self attention-based graph neural networks for hypergraphs

Self attention-based graph neural network for hypergraphs (Hyper-SAGNN) exploits the self-attention-based graph neural networks to refine the node features[8]. Hyper-SAGNN initializes node features by passing the adjacency matrix of the hypergraph $\mathbf{A} = \mathbf{H}\mathbf{H}^\top - \mathbf{D} \in \mathbb{R}^{n\times n}$ (defined differently from CMM and C3MM by discarding the self-loops) through a one-layer neural network, i.e.,

$$\mathbf{x}_i = \tanh(\mathbf{W}_{\text{enc}}\mathbf{a}_i + \mathbf{b}_{\text{enc}}) \text{ for } i = 1, 2, \dots, n, \tag{11}$$

where $\mathbf{a}_i \in \mathbb{R}^n$ are the columns of the adjacency matrix, and $\mathbf{W}_{\text{enc}}$ and $\mathbf{b}_{\text{enc}}$ are the learnable parameters in the encoder. Note that Hyper-SAGCN uses tanh as the default nonlinear activation function. Subsequently, Given a hyperlink e_p, HyperSAGNN incorporates two different ways (static and dynamic) to refine the features of the nodes within e_p, i.e.,

$$\begin{aligned} \mathbf{s}_i &= \tanh(\mathbf{W}_{\text{linear}}\mathbf{x}_i) \text{ for } v_i \in e_p \\ \mathbf{d}_i &= \tanh\Big(\sum_{\substack{v_i, v_j \in e_p \\ j \neq i}} \alpha_{ij}\mathbf{W}_{\text{conv}}\mathbf{x}_j\Big) \end{aligned} \tag{12}$$

where α_{ij} are the attention coefficients defined by

$$\alpha_{ij} = \frac{\exp\Big((\mathbf{W}_i^\top\mathbf{x}_i)^\top(\mathbf{W}_j^\top\mathbf{x}_j)\Big)}{\sum_{v_k\in e_p}\exp\Big((\mathbf{W}_i^\top\mathbf{x}_i)^\top(\mathbf{W}_k^\top\mathbf{x}_k)\Big)}, \tag{13}$$

and $\mathbf{W}_{\text{linear}}$ and $\mathbf{W}_{\text{conv}}$ are the learnable parameters in the static and dynamic neural networks, respectively. Therefore, the feature vector for e_p through a mean pooling function is given by

$$\mathbf{y}_p = \frac{1}{|e_p|}\sum_{v_i\in e_p}(\mathbf{s}_i - \mathbf{d}_i)^{*2}, \tag{14}$$

where the subscript $*2$ denotes the Hadamard power (element-wise power). The final scoring function is same as (10). It has been shown that HyperSAGNN does not perform well on relatively sparse hypergraphs such as metabolic networks.

Neural hyperlink predictor

Neural hyperlink predictor (NHP) shares a similar structure with Hyper-SAGNN, but employs a

new maximum minimum-based pooling function which can adaptively learn weights in a task-specific manner and include more prior knowledge about the nodes[9]. Similar to NVM, NHP initializes node features by performing Node2Vec on the clique-expanded graph. Suppose that the feature vector of node v_i is $\mathbf{x}_i$. Then NHP refines the features with a traditional graph neural network on each clique corresponding to a hyperlink in the original hypergraph. Given a hyperlink e_p, NHP computes

$$\tilde{\mathbf{x}}_i = \text{ReLU}(\mathbf{W}_{\text{conv1}}\mathbf{x}_i + \sum_{\substack{v_i, v_j \in e_p \\ j \neq i}} \mathbf{W}_{\text{conv2}}\mathbf{x}_j), \tag{15}$$

where $\mathbf{W}_{\text{conv1}}$ and $\mathbf{W}_{\text{conv2}}$ are the learnable parameters in the graph neural network. Note that NHP uses ReLU as the default nonlinear activation function. Subsequently, NHP uses a maximum minimum-based pooling function to compute hyperlink features, i.e.,

$$(\mathbf{y}_p^{(\text{maxmin})})_j = \max_{v_i \in e_p}\{(\tilde{\mathbf{x}}_i)_j\} - \min_{v_i \in e_p}\{(\tilde{\mathbf{x}}_i)_j\} \text{ for } j = 1, 2, \ldots, d_{\text{conv}}, \tag{16}$$

where d_{conv} denotes the hidden dimension of the graph neural network. The final scoring function is same as (10). NHP has the same issues with NVM, i.e., using Node2Vec on the clique-expanded graph could lead to a loss of higher-order structural information with higher computational costs.

Supplementary Note 2. CHESHIRE

Difference between CHESHIRE and NHP

NHP is a state-of-the-art algorithm for hyperlink prediction. Although CHESHIRE and NHP share a similar deep neural network architecture, CHESHIRE differs from NHP in the following aspects:
1. In the feature initialization step, NHP initializes node features by performing Node2Vec on the expanded graph. There are two limitations: (1) decomposing a hypergraph to a graph will result in a loss of higher-order structural information; and (2) Node2Vec is extremely expensive for large dense graphs since its time and memory dependencies on the graph's branching factor b (the number of children at each node) are $\mathcal{O}(b^2)$[10]. On the other hand, CHESHIRE generates node features by simply passing the incidence matrix through a one-layer neural network. The incidence matrix encodes all the higher-order topological attributes of the hypergraph, which can provide

more accurate initial node features with less computaional costs.

2. In the feature refinement step, NHP uses the traditional graph neural networks to refine node features, while CHESHIRE uses the highly sophisticated CSGCN. CSGCN exploits the Chebyshev polynomial expansion and spectral graph theory to learn the localized spectral filters which can extract local and composite features on graphs that encode complex geometric structures[11].

3. In the pooling step, NHP uses a new maximum minimum-based pooling function which can adaptively learn weights in a task-specific manner and include more prior knowledge about the nodes[9]. In addition to the maximum minimum-based pooling function, CHESHIRE incorporates a Frobenius norm-based pooling function, which is efficient at separating boundaries of the hyperlink feature space in learning hyperlink features[12].

4. Other than these major steps, CHESHIRE also utilizes advanced deep learning techniques including graph normalization[13] and alpha dropout[14] to smooth the learning process.

Complexity analysis

We analyze the computational complexity of CHESHIRE as follows. First, generating node features by passing the incidence matrix through a one-layer neural network takes $\mathcal{O}(nmd_{\text{enc}})$ time, where n and m are the total number of nodes and hyperlinks, respectively. During the feature refinement, the computational complexity of graph normalization and CSGCN are given by $\mathcal{O}(n_{\text{c}}d_{\text{enc}})$ and $\mathcal{O}(n_{\text{c}}m_{\text{c}}d_{\text{enc}}d_{\text{conv}}K)$, respectively. Here, n_{c} and m_{c} are the total number of nodes and edges in the disjoint graph (where $n_{\text{c}} = \sum_{p=1}^{m} |e_p|$ and $m_{\text{c}} = \sum_{p=1}^{m} \frac{1}{2}|e_p|(|e_p| - 1)$). We ignore the computational complexity for the alpha dropout layer since it is negligible. The final scoring layer including the Frobenius norm-based and the maximum minimum-based pooling functions takes $\mathcal{O}(n_{\text{c}}d_{\text{conv}} + md_{\text{conv}})$ time. Note that CSGCN is the most expensive component in CHESHIRE, which determines the overall time complexity.

Furthermore, we compared the running time of CHESHIRE with C3MM and NHP on the five largest GEMs (based on the number of reactions) from the BiGG database. We did not consider NVM because of its poor performance in internal validation. The testing GEMs include Recon3D (*Homo sapiens*), iCHOv1 (*Cricetulus griseus*), iLB1027_lipid (*Phaeodactylum tricornutum CCAP*

1055/1), iCHOv1_DG44 (*Cricetulus griseus*), and RECON1 (*Homo sapiens*). The running time is computed based on the first set of internal validation in a Mactonish machine with Apple M1 Pro chip and 32 GB memory. As shown in Supplementary Table 4, among all the three methods, CHESHIRE is the most computationally efficient method in predicting missing reactions.

Supplementary Note 3. Data and resources

BiGG models

The 108 BiGG models used in the internal validation were downloaded from the BiGG database (`http://BiGG.ucsd.edu`) in January 2022. Biomass reaction, exchange reactions, demand reactions and sink reactions were removed in each GEM before gap-filling as these types of reactions do not represent knowledge gaps.

Construction of BiGG universal reaction pool

The universal BiGG reaction database was downloaded from the BiGG database (`http://BiGG.ucsd.edu`). Biomass, exchange, demand, and sink reactions were removed. Reactions involving compartments other than cytosol, periplasm, and extracellular space were also removed. We further removed reactions with empty reaction names and excluded two reactions with imbalanced stoichiometry of carbon atom (FPGS_tm and SUCptspp_1). Finally, we excluded reactions whose identifiers start with letter ”r” and follow by digital numbers, all of which are derived from non-microbial GEMs. The resulting BiGG database contains 10,393 metabolites (unique IDs) and 16,337 reactions (unique IDs). We found that 2.45% of metabolite IDs have ambiguous names, i.e., names associated with more than one metabolite IDs. Similarly, 2.01% of reactions have ambiguous reaction formula, i.e., the same reactions associated with more than one identifier. Since the duplication of metabolites and reactions only inflate the entire database slightly, we did not further curate the BiGG universal database to resolve these inconsistencies.

Construction of BiGG genus-specific reaction pools

To find BiGG reactions that belong to a given taxonomic level, we used 818 AGORA[15] models

and their full taxonomy as a scaffold to map information. These models and their taxonomic information were downloaded from the Virtual Metabolic Human (VMH) database (https://www.vmh.life). We chose to build genus-specific reaction pools because the mean number of AGORA models per species (1.34; 611 species) is too few compared to that per genus (3.60; 227 genera). Since the namespace of VMH is different from that of BiGG, we mapped the reaction identifiers between the two databases by individually mapping reactions of each database to MetaNetX (https://www.metanetx.org), Seed (https://modelseed.org), and KEGG (https://www.genome.jp/kegg/). The mapping files were also downloaded from the BiGG and VMH databases. For each of the 227 VMH genera, we built its specific BiGG reaction database by aggregating all reactions in the BiGG universal databases if they were found in the VMH database and associated with a taxonomy.

Fermentation metabolite test data

The dataset contains 24 bacterial genomes (Supplementary Table 1) and the measurement of 9 fermentation products (acetic acid, butyric acid, ethanol, formic acid, lactic acid, butanol, propionaic acid, succinic acid and acetone) in the culture media. The genomes and fermentation data have been compiled by Zimmermann *et al.*[16] and released to the public (assessible from https://github.com/jotech/gapseq).

Amino acid secretion test data

The amino acid profile test was performed using data from a public study[17]. The dataset contains 25 bacterial genomes (Supplementary Table 2) and their associated 20 amino acid secretion profiles (histidine, isoleucine, leucine, lysine, methionine, phenylalanine, threonine, tryptophan, valine, alanine, asparagine, aspartic acid, glutamic acid, serine, arginine, cysteine, glutamine, glycine, proline, and tyrosine). The genomes were downloaded from The National Center for Biotechnology Information (NCBI) and the amino acid production profiles were obtained by personal communications with the corresponding author, Dr. Christian Kost.

Substrate utilization test data

The experimental substrate utilization tests were performed for growth of 5 bacterial species (Supplementary Table 3) using Biolog phenotype arrays[18]. The bacterial genomes and their test results have been compiled and made publicly available by a previous study[19] (accessible from `https://github.com/cdanielmachado/carveme`). All 5 species were tested for their utilization of carbon and nitrogen sources, except for *P. aeruginosa* PAO1 whose nitrogen source test was missing. *E. coli* str. K-12 substr. MG1655, *B. subtilis* 168, and *R. solanacearum* GMI1000 were additionally tested for their utilization of phosphorus and sulphur sources.

Gene essentiality test data

The essential and non-essential genes for 5 bacterial species (Supplementary Table 3) have been compiled and made publicly available by a previous study[19] (accessible from `https://github.com/cdanielmachado/carveme`).

Supplementary Note 4. Internal validation

Hyperparameter selection

We intended to fairly compare CHESHIRE with other approaches including NHP, C3MM, and NVM during internal validation. Similar as CHESHIRE, NHP and NVM are also not sensitive to their hyperparameters. We set the Node2Vec feature dimension to 256, which is consistent with the encoder dimension in CHESHIRE. The walk length and the number of walks per node were set to 80 and 10 in Node2Vec (default values in the Node2Vec Python package[20]), respectively. Additionally, we set the feature dimension of the graph neural network in NHP to 128, which is also consistent with the dimension of CSGCN in CHESHIRE. The learning rate of NHP and NVM is set to 0.01. For C3MM, we used the same latent space dimension 30 as used in the C3MM paper[7].

Threshold scores

We used a threshold score of 0.5 to determine whether an unseen reaction is true or false in Fig. 2. However, different threshold scores may lead to different performances of a model. Therefore, we

selected two reasonable threshold scores other than 0.5 in evaluating the performances of all the machine learning-based algorithms (CHESHIRE, NHP, NVM, and C3MM) over 108 BiGG GEMs. In particular, we used the mean and the median of all the unseen reactions' scores as the threshold score. Under the same settings used in Fig. 2a-d, we found that CHESHIRE still significantly outperforms the other machine learning-based methods in all the evaluation metrics (except for AUROC since it is independent of threshold scores) for the both threshold scores (Fig. 1). The mean threshold score gives rise to similar outcomes as in Fig. 2b-d (Supplementary Figure 1a-c), while the median threshold score results in similar Recall and Precision distributions (Supplementary Figure 1d-f). In fact, according to Fig. 2a, the plot of AUROC also indicates that CHESHIRE is the most robust algorithm to the threshold score.

Negative sampling strategies

Negative sampling is critically important in hyperlink prediction. Different negative sampling strategies may lead to different performances of a model. Here we considered a general negative sampling strategy. Suppose that we have a hypergraph $\mathcal{H} = \{\mathcal{V}, \mathcal{E}\}$ that captures a metabolic network. For each (positive) hyperlink $e \in \mathcal{E}$, we generate a corresponding negative hyperlink f, where $\alpha \times 100\%$ of the nodes in f are from e and the remaining are from $\mathcal{V} - e$ (the set of nodes that are not in e). The number α controls the genuineness of the negative reactions. Higher values of α indicate that the negative reactions are more close to the true. In Fig. 2, we used $\alpha = 0.5$ to sample negative reactions. In order to test the sensitivity of CHESHIRE to the negative sampling strategy, we further used $\alpha = 0.2$ and $\alpha = 0.8$ in evaluating the performances of all the machine learning-based algorithms (CHESHIRE, NHP, NVM, and C3MM) over 108 BiGG GEMs. We found that CHESHIRE still significantly outperforms the other machine learning-based methods in all the evaluation metrics for the both values of α (Supplementary Figure 2). When $\alpha = 0.2$, the negative reactions are more random, which enables all the algorithms to distinguish fake reactions easily (Supplementary Figure 2a-d). When $\alpha = 0.8$, the negative reactions are more close to the true, so it becomes difficult for the algorithms to distinguish negative reactions (Supplementary Figure 2e-h). Interestingly, C3MM outperforms NHP for all the evaluation metrics under this setting.

Negative sampling ratios

The negative sampling ratio between positive and negative reactions would also affect the performance of a model. In Fig. 2, we augmented the positive reactions by negative samples in a 1:1 ratio. We here changed the ratio to 1:2 and 1:3 in evaluating the performances of all the machine learning-based algorithms (CHESHIRE, NHP, NVM, and C3MM) over 108 BiGG GEMs. We found that CHESHIRE still significantly outperforms the other machine learning-based methods in all the evaluation metrics for the both negative sampling ratios (Supplementary Figure 3). Precision is most affected by the negative sample size for all the algorithms. On the other hand, AUROC and Recall behave similarly when increasing the negative sample size for the deep learning-based algorithms (NVM, NHP, and CHESHIRE).

Supplementary Note 5. External validation

Generation of GEMs

All draft GEMs were reconstructed using the standard CarveMe[19] or ModelSEED[21] pipelines. Only growth phenotypes were used for the built-in gap-filling algorithm in each pipeline. To fill the gaps in a given draft GEM, we first selected candidate BiGG or ModelSEED reactions whose confidence scores are equal to or greater than 0.9995 and then ranked them by their similarity scores, from lowest to highest. The top 200 reactions were iteratively added to the draft GEM. For each added reaction, we tested whether this reaction led to increased biomass flux, which indicates the establishment of energy-generating cycles (EGCs). EGCs are thermodynamically infeasible energy-generating cycles, which are capable of charging energy molecules without nutrient consumption. We used the method developed by Fritzemeier *et al.*[22] to detect EGCs. Briefly, we created 17 energy dissipation reactions and maximized the flux of one reaction at a time while prohibiting all influx into the model. These dissipation reactions correspond to 17 different types of energy metabolites: ATP (Adenosine Triphosphate), CTP (Cytidine Triphosphate), GTP (Guanosine Triphosphate), UTP (Uridine Triphosphate), ITP (Inosine Triphosphate), NADH (Reduced Nicotinamide Adenine Dinucleotide), NADPH (Reduced

Nicotinamide Adenine Dinucleotide Phosphate), FADH2 (Reduced Flavin Adenine Dinucleotide), FMNH2 (Reduced Flavin Mononucleotide), Q8H2 (Ubiquinol 8), Mql8 (Menaquinol 8), Mql6 (Menaquinol 6), Mql7 (Menaquinol 7), 2Dmmql8 (2-Demethylmenaquinol 8), AcCoA (Acetyl-CoA), L-Glutamate, and proton. Any non-zero flux through one of the 17 dissipation reactions indicates the presence of EGC that can generate the energy metabolite associated with the dissipation reaction. If the added reaction is reversible, we resolved the detected EGCs by changing its flux bounds: Its flux was restricted to be non-positive or non-negative if the reaction has a positive or negative flux in the EGC test. If the added reaction is irreversible, we skipped this reaction. For the fermentation metabolite test where bacteria were grown in anaerobic conditions, we skipped any reaction that contains oxygen as a reactant or product. We also excluded reactions that increased biomass flux over the known maximum growth rate of bacteria (2.81 1/hour, equivalent to 15 min/generation). This process continued until 200 reactions were added.

The standard reaction selection criteria described above have been used throughout this study, with the exception of the cofactor analysis presented below. The goal of cofactor analysis is to investigate the possibility of including cofactors as additional selection criteria, thereby further reducing the number of candidate reactions to be added. Here we focused on 15 cofactors, including ATP, CTP, GTP, UTP, ITP, NADPH, NADH, FADH2, FMNH2, Q8H2, Mql8, Mql6, Mql7, 2Dmmql8, and AcCoA. After applying the standard selection criteria based on confidence and similarity scores, we ranked these cofactors according to the number of reactions involving them in the draft GEM, from highest to lowest. We next tested four additional selection criteria to prioritize addition of reactions involving different cofactors: (1) excluding reactions that involve any of the above cofactors (NoCF); (2) excluding reactions that involve any cofactor except the top one identified above (Top1); (3) excluding reactions that involve any cofactor except the top two identified above (Top2); and (4) excluding reactions that involve any cofactor except the top three identified above (Top3). After excluding specified reactions, we added the remaining candidate reactions one at a time according to their similarity scores until a given number of reactions were added (50, 100 or 200). The results of this cofactor analysis are shown in Supplementary Figure 9.

Culture media compositions

The culture media compositions used for growth simulations were determined to reproduce the experimental conditions under which phenotypes were measured. While the dataset of fermentation product test result from multiple experiments whose culture media can vary, we followed the same strategy as described in Zimmermann *et al.*[16] and assumed that all experiments were performed under the same growth medium. We further adopted the fermentation test medium composition and their maximally allowed fluxes developed in the same study (accessible from https://github.com/Waschina/gapseqEval). For the amino acid secretion test, M9 minimal medium (with glucose) was used. Glucose has a maximum uptake rate of 10 mmol/gDW/h and all other compounds in the medium were unconstrained. For the substrate utilization test, GEMs were also constrained to the same M9 minimal medium, where the default sources of carbon, nitrogen, sulfur and phosphorus are glucose, ammonia, sulfate, and phosphate, respectively. For *Shewanella oneidensis*, the default carbon source is DL-lactate. To simulate growth on each substrate in Biolog arrays, the default source with the same type of the substrate (i.e., carbon, nitrogen, sulfur, and phosphorus) in the M9 minimal medium was replaced with the substrate. The maximum uptake rate for all Biolog substrates is 10 mmol/gDW/h and all other compounds in the M9 medium are unconstrained. We downloaded the M9 recipe from the github repository of CarveMe (accessible from `https://github.com/cdanielmachado/carveme`). For gene essentiality test, the culture media compositions were available from the same github repository: M9 minimal medium (with glucose) for *E. coli*, M9 minimal medium (with succinate) for *P. aeruginosa*, LB medium for *B. subtilis* and *S. oneidensis*, and complete medium (all compounds with exchange reactions are allowed to be uptaken) for *M. genitalium*. All compounds in the culture media were unconstrained.

Simulations of metabolic phenotypes

We adopted a similar strategy as used in Zimmermann *et al.*[16] to compute outflux values of secreted metabolites. For each GEM, we ran parsimonious Flux Balance Analysis (pFBA[23]) to avoid nutrient influxes that do not contribute to biomass and used pFBA solution to constrain import fluxes. Flux variability analysis[24] was applied to predict the maximum secretion fluxes of those metabolites under the constraint of maximum growth rate. Metabolites with a normalized

outflow (secretion flux divided by biomass) larger than 10^{-5} were considered as produced by the GEM. Therefore, our algorithm classified each metabolite as being produced or not produced by the GEM, which can be directly compared to the observed data.

We examined the ability of a draft GEM and its gap-filled version to produce a comprehensive list of 236 metabolites with BiGG IDs (`https://raw.githubusercontent.com/canc1993/cheshire-gapfilling/main/data/fermentation/substrate_exchange_reactions.csv`). This list was originally published by Zimmermann *et al.*[16]. For those metabolites which can only be produced by gap-filled GEMs, we determined and output the essential reactions needed for the production phenotypes using the algorithm specified in "Causal reaction inference in Supplementary Note 5".

We used Flux Balance Analysis (FBA)[25] to simulate bacterial growth on each substrate in Biolog phenotype arrays. The medium for each substrate was developed using the approach described in Culture Media Compositions. We used the function *single_gene_deletion* from the COBRApy package to simulate the effects of gene deletions on the growth phenotype. For both tests, a growth phenotype was considered positive if the growth rate was at least 0.01 h^{-1}.

Causal reaction inference

For any metabolite secreted by a gap-filled GEM but not by its corresponding draft GEM, we used Mixed Integer Linear Programming (MILP) to identify the minimum set of reactions added during gap-filling that enable the experimentally observed phenotype (Fig. 3a). The flux activity of each predicted reaction was described by a binary variable A under two linear constraints: (1) $f - f_{\text{min}}A \geq 0$ and (2) $f - f_{\text{max}}A \leq 0$, where f represents the flux of the reaction, and f_{min} and f_{max} were set to -1000 and 1000 respectively (i.e., the default lower and upper bounds of exchange reactions). Therefore, the reaction has unconstrained flux ($f \in [-1000, 1000]$) if $A = 1$ and carries zero flux ($f = 0$) if $A = 0$. Then we minimized the sum of all binary indicator variables under the constraint that the secretion flux of the metabolite is positive (a threshold of 0.1 was used). The minimal sum indicates the minimum number of reactions needed to gap-fill the draft GEM to

produce the metabolite and the identities of these key reactions can be obtained accordingly.

Enzymatic functional class of reactions

The enzymatic functional class of BiGG reactions was systematically extracted from their reaction names. We searched for keywords that end with “ase” and manually removed off-target hits (e.g., release). We further added two classes of reactions that may not be enzymatically catalyzed: (1) transport reactions if their names contain any of the following keywords (“transport”, “secretion”, “excretion”, “symport”, “antiport”, “uniport”, “uptake”, “efflux” and “diffusion”) and (2) formation/degradation reactions if their names contain the keyword “formation/degradation.”

Supplementary Table 1. Bacterial genomes used in our external validation for testing fermentation products.

NCBI Assembly	Taxonomy
GCF_000005845.2	*Escherichia coli* str. K-12 substr. MG1655
GCF_000008345.1	*Cutibacterium acnes* KPA171202
GCF_000008545.1	*Thermotoga maritima* MSB8
GCF_000008765.1	*Clostridium acetobutylicum* ATCC 824
GCF_000011065.1	*Bacteroides thetaiotaomicron* VPI-5482
GCF_000011985.1	*Lactobacillus acidophilus* NCFM
GCF_000013285.1	*Clostridium perfringens* ATCC 13124
GCF_000020425.1	*Bifidobacterium longum* subsp. infantis ATCC 15697
GCF_000020605.1	*Eubacterium rectale* ATCC 33656
GCF_000022965.1	*Bifidobacterium animalis* subsp. lactis DSM 10140
GCF_000025885.1	*Aminobacterium colombiense* DSM 12261
GCF_000056065.1	*Lactobacillus delbrueckii* subsp. bulgaricus ATCC 11842
GCF_000143845.1	*Olsenella uli* DSM 7084
GCF_000144405.1	*Prevotella melaninogenica* ATCC 25845
GCF_000160535.1	*Prevotella bergensis* DSM 17361
GCF_000173975.1	*Anaerobutyricum hallii* DSM 3353
GCF_000175255.2	*Zymomonas mobilis* subsp. mobilis ATCC 10988
GCF_000389635.1	*Clostridium pasteurianum* BC1
GCF_000392875.1	*Enterococcus faecalis* ATCC 19433
GCF_000469345.1	*Eubacterium ramulus* ATCC 29099
GCF_001456065.2	*Clostridium butyricum* KNU-L09
GCF_001561955.1	*Anaerotignum propionicum* DSM 1682
GCF_000162015.1	*Faecalibacterium prausnitzii* A2-165
GCF_000203855.3	*Lactobacillus plantarum* WCFS1

Supplementary Table 2. Bacterial genomes used in our external validation for testing amino acids secretions.

NCBI Assembly	Taxonomy
GCF_002895265.1	*Azospirillum brasilense* DSM 1690
GCF_900187015.1	*Serratia entomophila* DSM 12358
GCF_000009045.1	*Bacillus subtilis* 168
GCF_000046845.1	*Acinetobacter baylyi* ADP1
GCF_002055965.1	*Bacillus subtilis* 3610 ComIQ12L
GCF_000005845.2	*Escherichia coli* MG1655 DSM 18039
GCF_000750555.1	*Escherichia coli* BW25113
GCF_000009225.2	*Pseudomonas fluorescens* SBW25
GCF_000012265.1	*Pseudomonas fluorescnes* Pf-5
GCF_000007565.2	*Pseudomonas putida* KT2440
GCF_000196235.1	*Arthrobaacter nicotianae* DSM 20123
GCF_000971565.1	*Agrobacterium tumefaciens*
GCF_000007805.1	*Pseudomonas syringae* pv. tomato DC 3000
GCF_000012245.1	*Pseudomonas syringae* pv. tomato DSM 50315
GCF_000454045.1	*Nocardia coeliaca*
GCF_001578185.1	*Bacillus simplex*
GCF_000196015.1	*Cupriavidus metallidurans*
GCF_000011645.1	*Bacillus licheniformis*
GCF_001591345.1	*Variovorax boronicumulans*
GCF_900187015.1	*Serratia ficaria*
GCF_002009195.1	*Bacillus megaterium* DSM 32
GCF_000237065.1	*Pseudomonas fluorescens* DSM 289
GCF_000016645.1	*Flavobacterium johnsoniae* DSM 2064
GCF_002303785.1	*Rahnella victoriana* DSM 27397
GCF_000023825.1	*Pedobacter heparinus* DSM 2366

Supplementary Table 3. Bacterial genomes used in our external validation for testing growth phenotypes and gene essentiality. *M. genitalium* G-37 was not used for growth phenotype and *R. solanacearum* GMI1000 was not used in gene essentiality test.

NCBI Assembly	Taxonomy
GCF_000005845.2	*Escherichia coli* str. K-12 substr. MG1655
GCF_000009045.1	*Bacillus subtilis* 168
GCF_000006765.1	*Pseudomonas aeruginosa* PAO1
GCF_000009125.1	*Ralstonia solanacearum* GMI1000
GCF_000146165.2	*Shewanella oneidensis* MR-1
GCF_000027325.1	*Mycoplasmoides genitalium* G-37

Supplementary Table 4. Computational time comparison (in second) for CHEHISRE, NHP, and C3MM on the five largest models from the BiGG database. The computational time is computed based on the first type of internal validation in a Mactonish machine with Apple M1 Pro chip and 32 GB memory.

BiGG Model	Recon3D	iCHOv1	iLB1027_lipid	iCHOv1_DG44	RECON1
C3MM	2,871.36	1,456.44	636.37	441.51	444.37
NHP	321.38	175.88	91.14	86.18	86.28
CHESHIRE	211.15	109.55	63.30	45.04	42.83

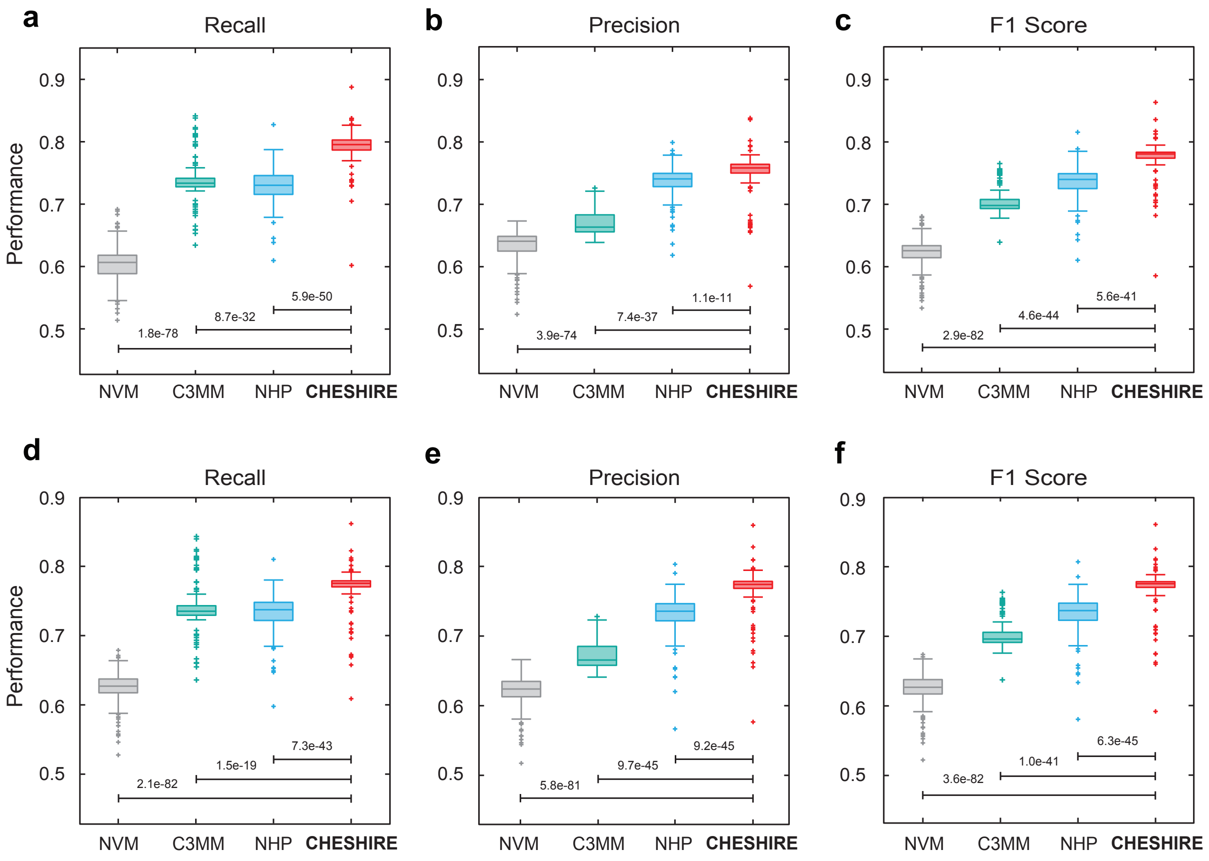

Supplementary Figure 1. Internal validation using artificially introduced gaps with mean and median threshold scores. a-c Boxplots of the performance metrics (Recall, Precision, and F1 score) calculated on 108 BiGG GEMs (each dot represents a GEM) for CHESHIRE vs. NHP, C3MM, and NVM using the mean threshold score. **d-f** Boxplots of the performance metrics (Recall, Precision, and F1 score) calculated on 108 BiGG GEMs (each dot represents a GEM) for CHESHIRE vs. NHP, C3MM, and NVM using the median threshold score. Each data point is the mean over 10 Monte Carlo runs. Boxplot: central line represents the median, box limits represent the first and third quartiles, and whiskers extend to the smallest and largest values or at most to 1.5× the interquartile range, whichever is smaller. Two-sided paired-sample t-test: exact p-values are provided. Source data are provided as a Source Data file.

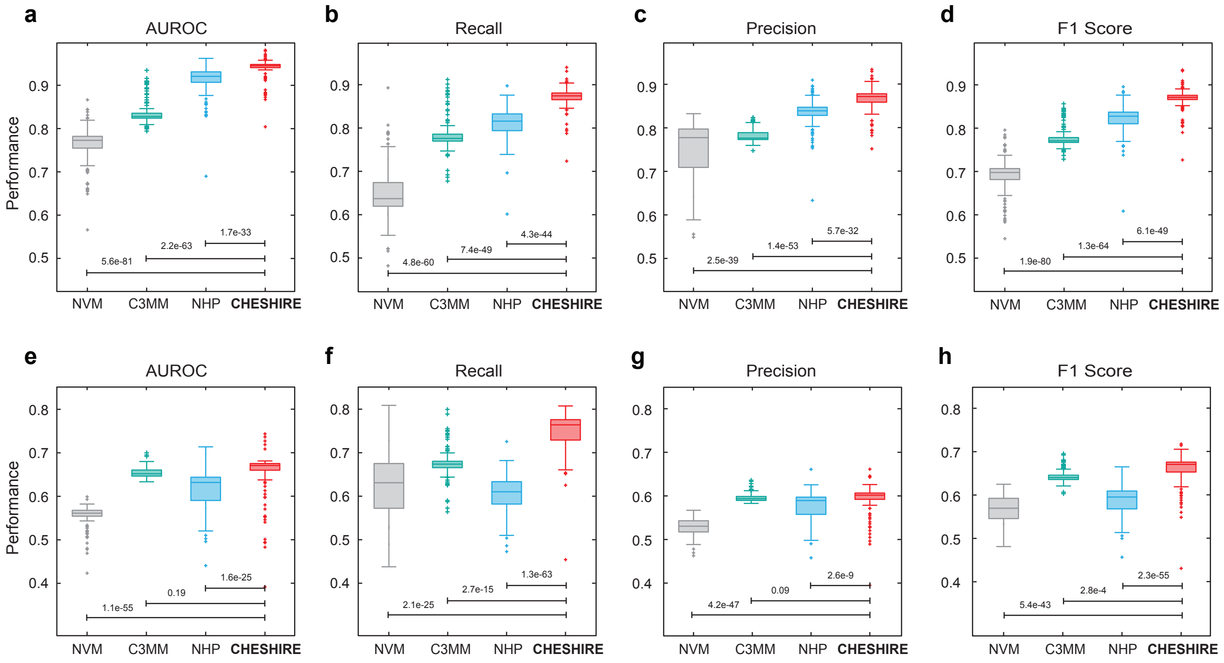

Supplementary Figure 2. Internal validation using artificially introduced gaps with different negative sampling strategies. a-d Boxplots of the performance metrics (AUROC, Recall, Precision, and F1 score) calculated on 108 BiGG GEMs (each dot represents a GEM) for CHESHIRE vs. NHP, C3MM, and NVM using the negative sampling strategy with $\alpha = 0.2$. **e-h** Boxplots of the performance metrics (AUROC, Recall, Precision, and F1 score) calculated on 108 BiGG GEMs (each dot represents a GEM) for CHESHIRE vs. NHP, C3MM, and NVM using the negative sampling strategy with $\alpha = 0.8$. Each data point is the mean over 10 Monte Carlo runs. Boxplot: central line represents the median, box limits represent the first and third quartiles, and whiskers extend to the smallest and largest values or at most to 1.5× the interquartile range, whichever is smaller. Two-sided paired-sample t-test: exact p-values are provided. Source data are provided as a Source Data file.

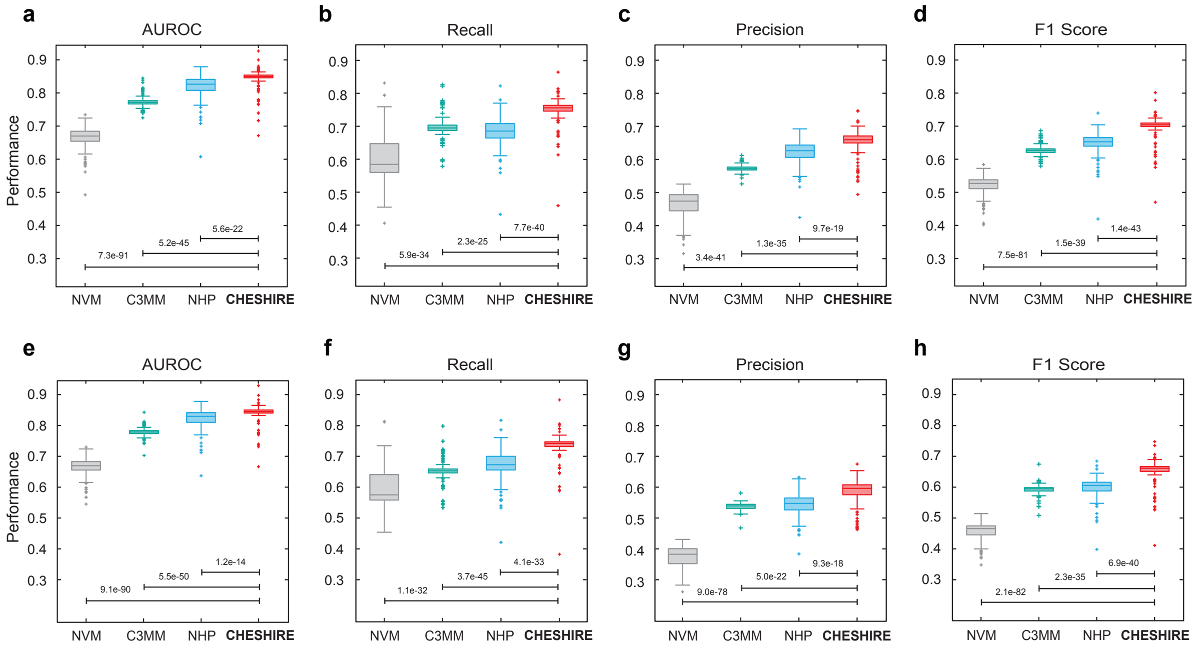

Supplementary Figure 3. Internal validation using artificially introduced gaps with different negative sampling ratio. a-d Boxplots of the performance metrics (AUROC, Recall, Precision, and F1 score) calculated on 108 BiGG GEMs (each dot represents a GEM) for CHESHIRE vs. NHP, C3MM, and NVM using 1:2 negative sampling ratio. **e-h** Boxplots of the performance metrics (AUROC, Recall, Precision, and F1 score) calculated on 108 BiGG GEMs (each dot represents a GEM) for CHESHIRE vs. NHP, C3MM, and NVM using 1:3 negative sampling ratio. Each data point is the mean over 10 Monte Carlo runs. Boxplot: central line represents the median, box limits represent the first and third quartiles, and whiskers extend to the smallest and largest values or at most to 1.5× the interquartile range, whichever is smaller. Two-sided paired-sample t-test: exact p-values are provided. Source data are provided as a Source Data file.

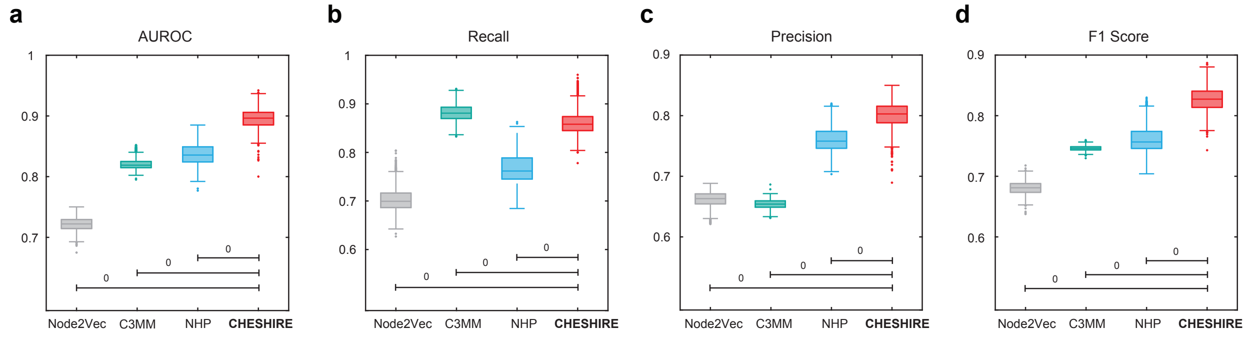

Supplementary Figure 4. Internal validation using artificially introduced gaps on AGORA GEMs of gut bacteria. a-d Boxplots of the performance metrics (AUROC, Recall, Precision, and F1 score) calculated on 818 AGORA GEMs (each dot represents a GEM) for CHESHIRE vs. NHP, C3MM, and NVM. Each data point is the mean over 10 Monte Carlo runs. Boxplot: central line represents the median, box limits represent the first and third quartiles, and whiskers extend to the smallest and largest values or at most to 1.5× the interquartile range, whichever is smaller. Two-sided paired-sample t-test: exact p-values are provided. Source data are provided as a Source Data file.

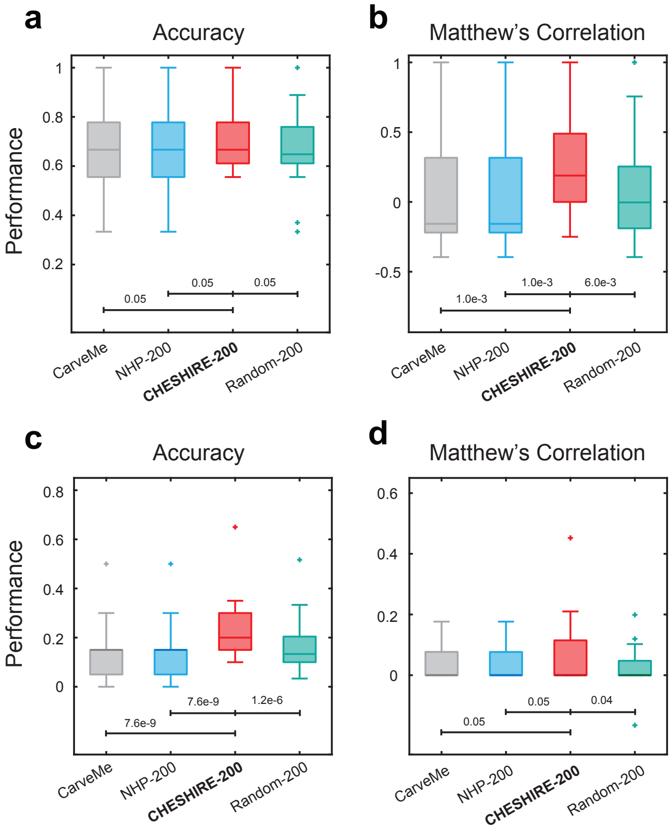

Supplementary Figure 5. External validation evaluated using overall accuracy and Matthew's correlation coefficients. a, b The fermentation metabolite test (24 bacterial GEMs). **c, d** The amino acid test (25 bacterial GEMs). Each dot represents a GEM. CarveMe: CarveMe-reconstructed GEMs; NHP-200: draft models plus 200 NHP-predicted missing reactions; CHESHIRE-200: draft models plus 200 CHESHIRE-predicted missing reactions; Random-200: draft models plus 200 randomly selected reactions (performance averaged over 3 Monte Carlo runs). Boxplot: central line represents the median, box limits represent the first and third quartiles, and whiskers extend to the smallest and largest values or at most to 1.5× the interquartile range, whichever is smaller. Two-sided paired-sample t-test: exact p-values are provided. Source data are provided as a Source Data file.

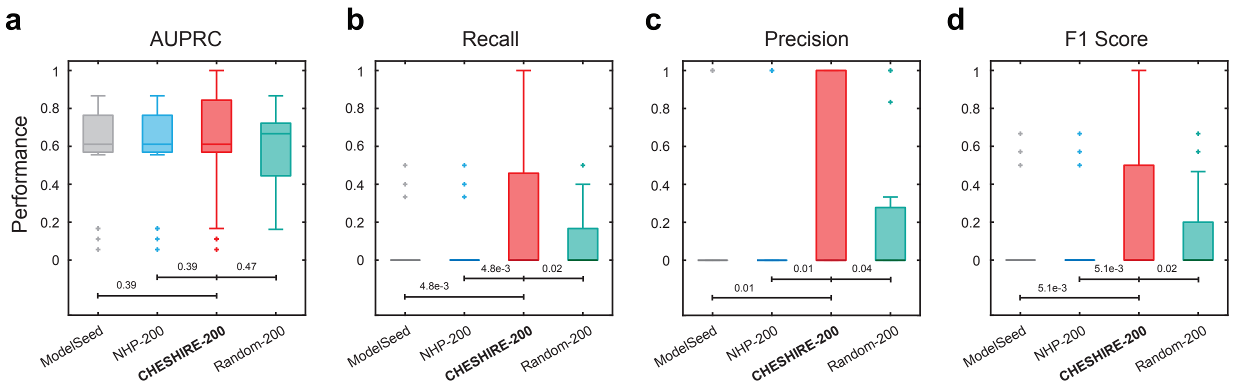

Supplementary Figure 6. The fermentation metabolite test (external validation) using ModelSEED-reconstructed draft GEMs. a-d Boxplots of the performance metrics (AUPRC, Recall, Precision, and F1 score) calculated on 24 bacterial GEMs for CHESHIRE-200 (draft models plus 200 CHESHIRE-predicted missing reactions) vs. ModelSEED (ModelSEED-reconstructed GEMs), NHP-200 (draft models plus 200 NHP-predicted missing reactions), and Random-200 (draft models plus 200 randomly selected reactions; performance averaged over 3 Monte Carlo runs). Boxplot: central line represents the median, box limits represent the first and third quartiles, and whiskers extend to the smallest and largest values or at most to 1.5× the interquartile range, whichever is smaller. Two-sided paired-sample t-test: exact p-values are provided. Source data are provided as a Source Data file.

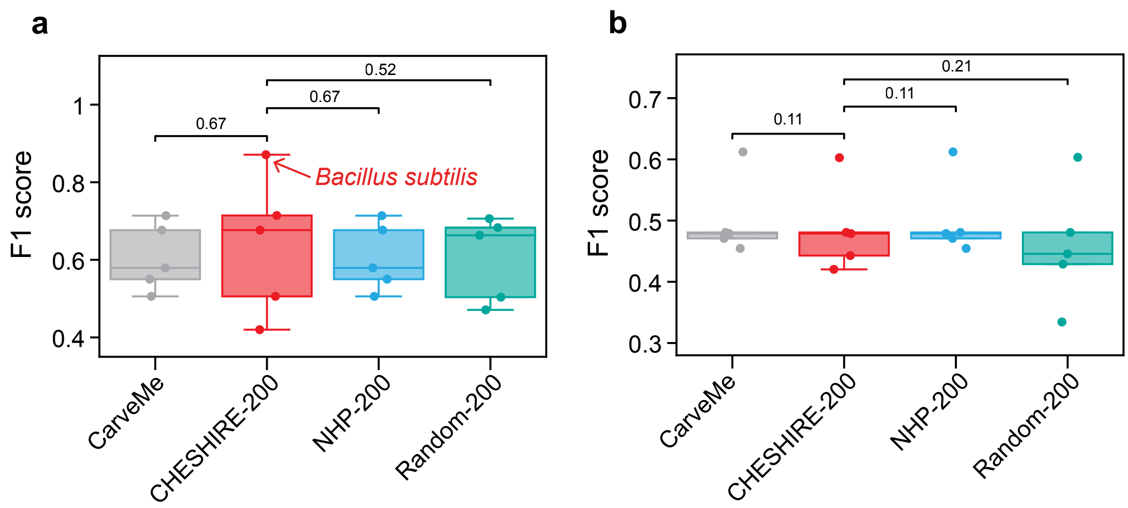

Supplementary Figure 7. Performance evaluation of CHESHIRE on 5 bacterial GEMs for filling the gaps in (a) growth phenotype and (b) gene essentiality. Each dot in a boxplot represents a GEM. CarveMe: draft models reconstructed from the CarveMe pipeline; CHESHIRE-200: draft models plus 200 missing reactions predicted by CHESHIRE; NHP-200: draft models plus 200 missing reactions predicted by NHP; Random-200: draft models plus 200 randomly selected reactions. Boxplot: central line represents the median, box limits represent the first and third quartiles, and whiskers extend to the smallest and largest values or at most to 1.5× the interquartile range, whichever is smaller. Two-sided paired-sample t-test: exact p-values are provided. Source data are provided as a Source Data file.

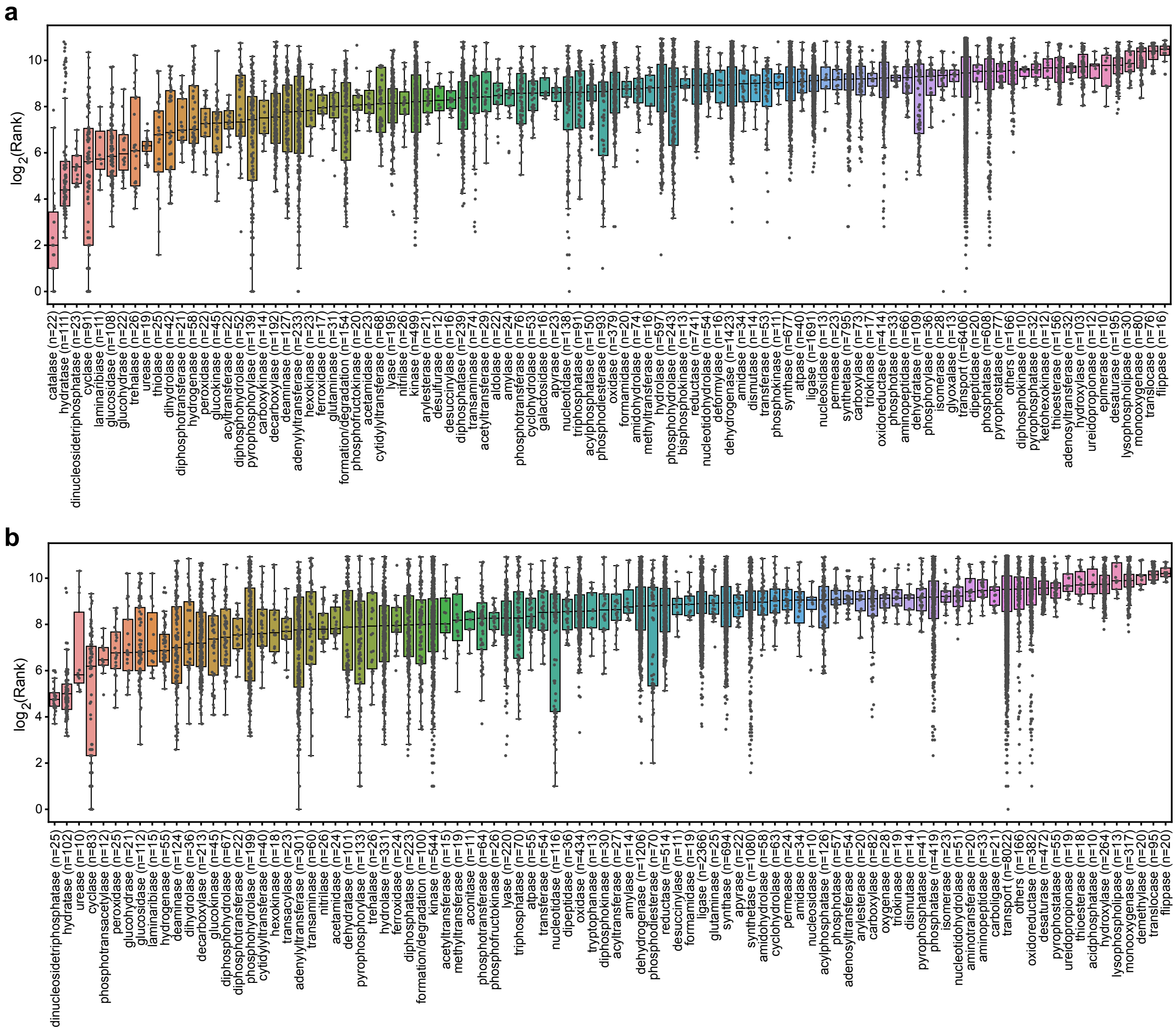

Supplementary Figure 8. Reaction rankings categorized by enzymatic functional classes. Each dot represents a specific reaction and all dots for each boxplot represent all reactions catalyzed by enzymes of a specific functional class. Panel **a** was drawn using reaction rankings from GEMs in the fermentation product test and panel **b** was drawn using reaction rankings from GEMs in the amino acid test. Boxplot: central line represents the median, box limits represent the first and third quartiles, and whiskers extend to the smallest and largest values or at most to 1.5× the interquartile range, whichever is smaller. Source data are provided as a Source Data file.

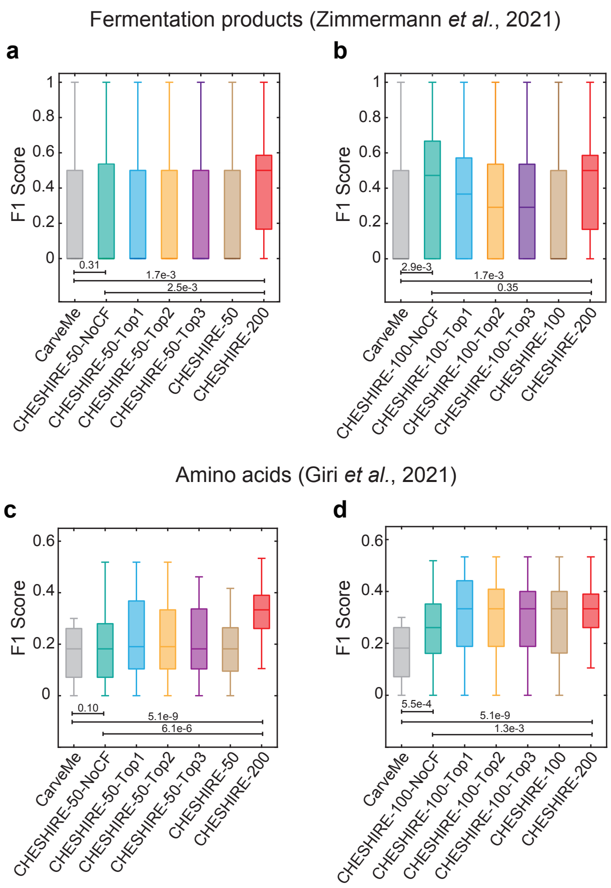

Supplementary Figure 9. Comparison of CHESHIRE performance across various strategies for prioritizing cofactor-containing reactions using the fermentation metabolite test (24 bacterial GEMs) in (a, b) and the amino acid test (25 bacterial GEMs) in (c, d). The tested strategies include: CarveMe - GEMs reconstructed using CarveMe; CHESHIRE-50/100/200 - draft models plus 50/100/200 CHESHIRE-predicted reactions (all cofactors allowed); CHESHIRE-50/100-NoCF - draft models plus 50/100 CHESHIRE-predicted reactions that involve none of the 15 specified cofactors (see Section 6.1); CHESHIRE-50/100-Top1/Top2/Top3 - draft models plus 50/100 CHESHIRE-predicted reactions that do not involve any of the 15 specified cofactors or involve only the top 1/2/3 cofactors with the highest prevalence in the draft GEMs. Boxplot: central line represents the median, box limits represent the first and third quartiles, and whiskers extend to the smallest and largest values or at most to 1.5× the interquartile range, whichever is smaller. Two-sided paired-sample t-test: exact p-values are provided. Source data are provided as a Source Data file.

Supplementary references

1. Vinay Satish Kumar, Madhukar S Dasika, and Costas D Maranas. Optimization based automated curation of metabolic reconstructions. *BMC Bioinform.* **8**, 1–16 (2007).
2. Ines Thiele, Nikos Vlassis, and Ronan MT Fleming. FastGapFill: Efficient gap filling in metabolic networks. *Bioinformatics* **30**, 2529–2531 (2014).
3. Nikos Vlassis, Maria Pires Pacheco, and Thomas Sauter. Fast reconstruction of compact context-specific metabolic network models. *PLoS Comput. Biol.* **10**, e1003424 (2014).
4. Muhan Zhang, Zhicheng Cui, Tolutola Oyetunde, Yinjie Tang, and Yixin Chen. Recovering metabolic networks using a novel hyperlink prediction method. *arXiv* (2016).
5. Muhan Zhang, Zhicheng Cui, Shali Jiang, and Yixin Chen. Beyond link prediction: Predicting hyperlinks in adjacency space. In *Proceedings of the 32th Conference on Artificial Intelligence*, 4430–4437 (2018).
6. IBM ILOG Cplex. V12. 1: User's manual for cplex. *IBM* **46**, 157 (2009).
7. Govind Sharma, Prasanna Patil, and M Narasimha Murty. C3MM: Clique-closure based hyperlink prediction. In *Proceedings of the 29th International Conference on International Joint Conferences on Artificial Intelligence (IJCAI)*, 3364–3370 (2020).
8. Ruochi Zhang, Yuesong Zou, and Jian Ma. Hyper-sagnn: A self-attention based graph neural network for hypergraphs. In *Proceedings of the 8th International Conference on Learning Representations (ICLR)* (2020).
9. Naganand Yadati et al. NHP: Neural hypergraph link prediction. In *Proceedings of the 29th ACM International Conference on Information and Knowledge Management (CIKM)*, 1705–1714 (2020).
10. Tiago Pimentel, Rafael Castro, Adriano Veloso, and Nivio Ziviani. Efficient estimation of node representations in large graphs using linear contexts. In *Proceedings of the 28th International Joint Conference on Neural Networks (IJCNN)*, 1–8 (IEEE, 2019).

11. Michaël Defferrard, Xavier Bresson, and Pierre Vandergheynst. Convolutional neural networks on graphs with fast localized spectral filtering. *Adv. Neural Inf. Process. Syst.* **29**, 3844–3852 (2016).

12. Caglar Gulcehre, Kyunghyun Cho, Razvan Pascanu, and Yoshua Bengio. Learned-norm pooling for deep feedforward and recurrent neural networks. In *Joint European Conference on Machine Learning and Knowledge Discovery in Databases (ECML PKDD)*, 530–546 (Springer, 2014).

13. Tianle Cai et al. GraphNorm: a principled approach to accelerating graph neural network training. In *Proceedings of the 38th International Conference on Machine Learning (ICML)*, 1204–1215 (PMLR, 2021).

14. Günter Klambauer, Thomas Unterthiner, Andreas Mayr, and Sepp Hochreiter. Self-normalizing neural networks. In *Proceedings of the 31st International Conference on Neural Information Processing Systems (NeurIPS)*, 972–981 (2017).

15. Stefanía Magnúsdóttir et al. Generation of genome-scale metabolic reconstructions for 773 members of the human gut microbiota. *Nat. Biotechnol.* **35**, 81–89 (2017).

16. Johannes Zimmermann, Christoph Kaleta, and Silvio Waschina. gapseq: informed prediction of bacterial metabolic pathways and reconstruction of accurate metabolic models. *Genome Biol.* **22**, 1–35 (2021).

17. Samir Giri et al. Metabolic dissimilarity determines the establishment of cross-feeding interactions in bacteria. *Curr. Biol.* **31**, 5547–5557 (2021).

18. WS Mauchline and CW Keevil. Development of the biolog substrate utilization system for identification of legionella spp. *Appl. Environ. Microbiol.* **57**, 3345–3349 (1991).

19. Daniel Machado, Sergej Andrejev, Melanie Tramontano, and Kiran Raosaheb Patil. Fast automated reconstruction of genome-scale metabolic models for microbial species and communities. *Nucleic Acids Res.* **46**, 7542–7553 (2018).

20. Elior Cohen. Node2vec (2022).

21. Samuel MD Seaver et al. The ModelSEED Biochemistry Database for the integration of metabolic annotations and the reconstruction, comparison and analysis of metabolic models for plants, fungi and microbes. *Nucleic Acids Res.* **49**, D575–D588 (2021).

22. Claus Jonathan Fritzemeier, Daniel Hartleb, Balázs Szappanos, Balázs Papp, and Martin J Lercher. Erroneous energy-generating cycles in published genome scale metabolic networks: identification and removal. *PLoS Comput. Biol.* **13**, e1005494 (2017).

23. Nathan E Lewis et al. Omic data from evolved *E. coli* are consistent with computed optimal growth from genome-scale models. *Mol. Syst. Biol.* **6**, 390 (2010).

24. I Thiele and S Gudmundsson. Computationally efficient flux variability analysis. *BMC Bioinform.* **11**, 1–3 (2010).

25. Jeffrey D Orth, Ines Thiele, and Bernhard Ø Palsson. What is flux balance analysis? *Nat. Biotechnol.* **28**, 245–248 (2010).
