# Supplementary material for: Teasing out missing reactions in genome-scale metabolic networks through hypergraph learning
Source: Nat Commun. 2023 Apr 25;14:2375. doi: 10.1038/s41467-023-38110-7 (PMC10130184; doi:10.1038/s41467-023-38110-7)
Supplement: Supplementary file 3 — Reporting Summary [file 41467_2023_38110_MOESM3_ESM.pdf]

nature portfolio

Corresponding author(s): Yang-Yu Liu
Last updated by author(s): Apr 6, 2023

Reporting Summary

Nature Portfolio wishes to improve the reproducibility of the work that we publish. This form provides structure for consistency and transparency in reporting. For further information on Nature Portfolio policies, see our Editorial Policies and the Editorial Policy Checklist.

Statistics

For all statistical analyses, confirm that the following items are present in the figure legend, table legend, main text, or Methods section.

n/a	Confirmed	
☐	☒	The exact sample size (*n*) for each experimental group/condition, given as a discrete number and unit of measurement
☐	☒	A statement on whether measurements were taken from distinct samples or whether the same sample was measured repeatedly
☐	☒	The statistical test(s) used AND whether they are one- or two-sided<br>*Only common tests should be described solely by name; describe more complex techniques in the Methods section.*
☒	☐	A description of all covariates tested
☐	☒	A description of any assumptions or corrections, such as tests of normality and adjustment for multiple comparisons
☐	☒	A full description of the statistical parameters including central tendency (e.g. means) or other basic estimates (e.g. regression coefficient) AND variation (e.g. standard deviation) or associated estimates of uncertainty (e.g. confidence intervals)
☐	☒	For null hypothesis testing, the test statistic (e.g. *F*, *t*, *r*) with confidence intervals, effect sizes, degrees of freedom and *P* value noted<br>*Give P values as exact values whenever suitable.*
☒	☐	For Bayesian analysis, information on the choice of priors and Markov chain Monte Carlo settings
☒	☐	For hierarchical and complex designs, identification of the appropriate level for tests and full reporting of outcomes
☐	☒	Estimates of effect sizes (e.g. Cohen's *d*, Pearson's *r*), indicating how they were calculated

Our web collection on statistics for biologists contains articles on many of the points above.

Software and code

Policy information about availability of computer code

Data collection	No software was used for data collection. Published datasets were obtained either by downloading them from supplementary materials or through personal communications.
Data analysis	All computational analysis was performed using customized Python (3.7) codes with cobrapy (0.22.1), cplex solver (12.8), joblib (1.1.0), numpy (1.21.2), optlang (1.5.2), pandas (1.3.2), torch (1.9.0), torch-geometric (1.7.2), torch-scatter (2.0.8), torch-sparse (0.6.11), and tqdm (4.62.1).<br>Computational code for Node2Vec can be found at https://github.com/eliorc/node2vec.<br>Computational code for CHESHIRE can be found at https://github.com/canc1993/cheshire-gapfilling.<br>Computational codes for C3MM and NHP were obtained from personal communication.

For manuscripts utilizing custom algorithms or software that are central to the research but not yet described in published literature, software must be made available to editors and reviewers. We strongly encourage code deposition in a community repository (e.g. GitHub). See the Nature Portfolio guidelines for submitting code & software for further information.

Data

Policy information about availability of data

All manuscripts must include a data availability statement. This statement should provide the following information, where applicable:

- Accession codes, unique identifiers, or web links for publicly available datasets
- A description of any restrictions on data availability
- For clinical datasets or third party data, please ensure that the statement adheres to our policy

All genome-scale metabolic models (GEMs) and associated phenotypic data are publicly available. The 108 BiGG GEMs and BiGG universal reaction database were downloaded from the BiGG database (http://bigg.ucsd.edu). The 818 AGORA models were downloaded from the Virtual Metabolic Human database (https://www.vmh.life). The external validation used four phenotypic datasets: (1) 9 fermentation metabolites produced by 24 bacterial species (Zimmermann et al., Genome Biology, 2021); 20 amino acids produced by 25 bacterial species (Giri, et. al., Current Biology, 2021); (3) utilization of various carbon-, nitrogen-, sulpher-, and phosphorus-substrates for growth of 5 bacterial species (Machado et al., Nucleic Acids Research, 2018); (4) lists of essential and non-essential genes of 5 bacterial species (Machoda et al., Nucleic Acid Resarch, 2018). See Supplementary Note 3 for details of these datasets. Source data generated by our computational analysis are provided with this paper.

Human research participants

Policy information about studies involving human research participants and Sex and Gender in Research.

Reporting on sex and gender	N/A
Population characteristics	N/A
Recruitment	N/A
Ethics oversight	N/A

Note that full information on the approval of the study protocol must also be provided in the manuscript.

Field-specific reporting

Please select the one below that is the best fit for your research. If you are not sure, read the appropriate sections before making your selection.

☒ Life sciences ☐ Behavioural & social sciences ☐ Ecological, evolutionary & environmental sciences

For a reference copy of the document with all sections, see nature.com/documents/nr-reporting-summary-flat.pdf

Life sciences study design

All studies must disclose on these points even when the disclosure is negative.

Sample size	No calculation of sample sizes were made since we used the publicly available datasets. For internal validation, we used all genome-scale metabolic models (GEMs) in the top two most curated GEM databases, including 108 BiGG models and 818 VMH models. For external validation, we employed a total of 55 GEMs that have publicly available phenotypic data, including secretion of fermentation metabolites (n=24), secretion of amino acids (n=25), and substrate utilization/gene essentiality (n=6). The consistent performance of our method through different datasets demonstrates that such sample sizes are sufficient.
Data exclusions	No GEM was excluded.
Replication	This study did not generate any new experimental data and instead reused previously published data. For internal validation, we performed random train test split and repeated the process over 10 Monte Carlo runs. External validations included the repetition of the prediction score for a candidate reaction 5 times, and the average score was calculated.
Randomization	We used a random train-test split of reactions in the internal validation. In the external validation, we added 200 random reactions to draft genome-scale metabolic models as controls.
Blinding	Blinding is not relevant for this study, as it does not use data from clinical case-control studies. However, blinding was simulated during the development of machine learning models, where data in the test sets were not visible during training.

Reporting for specific materials, systems and methods

We require information from authors about some types of materials, experimental systems and methods used in many studies. Here, indicate whether each material, system or method listed is relevant to your study. If you are not sure if a list item applies to your research, read the appropriate section before selecting a response.

Materials & experimental systems

n/a	Involved in the study
☒	☐ Antibodies
☒	☐ Eukaryotic cell lines
☒	☐ Palaeontology and archaeology
☒	☐ Animals and other organisms
☒	☐ Clinical data
☒	☐ Dual use research of concern

Methods

n/a	Involved in the study
☒	☐ ChIP-seq
☒	☐ Flow cytometry
☒	☐ MRI-based neuroimaging
